# Sensitivity and noise in THz electro-optic upconversion radiometers

**DOI:** 10.1038/s41598-020-65987-x

**Published:** 2020-06-10

**Authors:** Gabriel Santamaría-Botello, Zoya Popovic, Kerlos Atia Abdalmalak, Daniel Segovia-Vargas, Elliott R. Brown, Luis Enrique García Muñoz

**Affiliations:** 1 Signal Theory and Communications Department, Charles III University of Madrid, Madrid, Spain; 2 Department of Electrical, Computer, and Energy Engineering, University of Colorado Boulder, Boulder, CO USA; 3 Wright State University, Dayton, OH USA

**Keywords:** Optics and photonics, Optical physics, Nonlinear optics

## Abstract

This paper presents a study of noise in room-temperature THz radiometers that use THz-to-optical upconversion followed by optical detection of thermal radiation. Despite some undesired upconverted thermal noise, no noise is intrinsically introduced by efficient electro-optic modulation via a sum-frequency-generation process in high quality factor (Q) whispering-gallery mode (WGM) resonators. However, coherent and incoherent optical detection results in fundamentally different noise characteristics. The analysis shows that the upconversion receiver is quantum limited like conventional amplifiers and mixers, only when optical homodyne or heterodyne detection is performed. However, this type of receiver shows advantages as a THz photon counter, where counting is in the optical domain. Theoretical predictions show that upconversion-based room-temperature receivers can outperform state-of-the-art cooled and room-temperature THz receivers based on low-noise amplifiers and mixers, provided that a photon conversion efficiency greater than 1% is realized. Although the detection bandwidth is naturally narrow due to the highly resonant electro-optic modulator, it is not fundamentally limited and can be broadened by engineering selective optical coupling mechanisms to the resonator.

## Introduction

Millimeter-wave, terahertz and far-IR radiometers are used in radio-astronomy and astrophysics, as well as in earth sciences, since many important emission lines between 200 GHz and 2.5 THz can be monitored for pollution, meteorology, and atmospheric modeling, as reviewed in^1^. In environmental atmospheric sensing, e.g., instruments were built to detect organic acid C10 line in the atmosphere at 700 GHz^2^, to observe cloud particle size and ice water from 240 to 850 GHz^3^, and for OH measurements in the stratosphere and mesosphere from space at 2.5 THz. Space THz instruments for molecular line spectroscopy from about 100 GHz to 1.5 THz is reviewed in^4^. Other direct-detection, pre-amplified and heterodyne imaging radiometers were designed to observe millimeter-wave and THz bands for various applications such as concealed weapon detection and plume imaging^5–9^. A recent review of cryogenic ground-based and airborne far-IR radio-astronomy instrumentation in the frequency range from 300 GHz to 10 THz is given in^10^.

**Figure 1:**
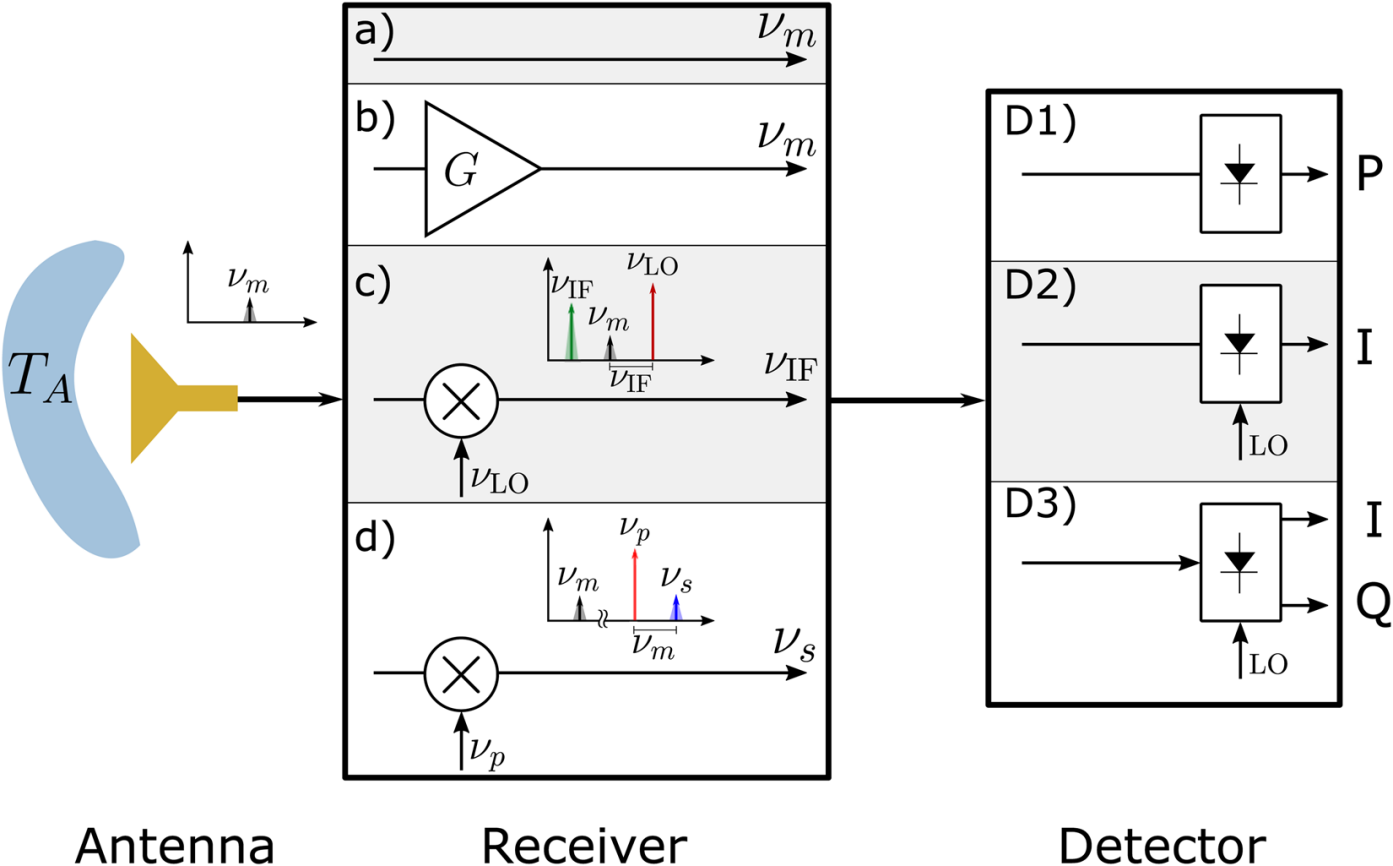
General radiometer receiver and detector schemes. The power of the THz radiation collected by the antenna, represented here by some temperature *T*_*A*_, can be directly received and detected with direct detection as in case (D1), homodyne (D2) or heterodyne detection (D3). To improve the SNR, the received radiation is often preamplified as in case (**b**), down-converted as in case (**c**) or can be upconverted as in case (**d**).

Despite successful efforts such as the ones listed above, low-noise high-sensitivity detection in the THz frequency range remains challenging since conventional receivers are either non-existent or less sensitive than their microwave and optical counterparts. Coherent receivers are used in interferometry due to the requirement for preserving the phase information. However, even when only power spectral information is needed, heterodyne receivers are often used, since backend digital spectrometers can resolve the baseband spectrum and are a simpler approach than using complex filter banks before an incoherent detector^11^. Figure 1 illustrates the various types of receivers, where detectors are shown on the right and can respond to the power of the incident radiation (D1), or field components in phase and/or in quadrature to a local oscillator, depending on whether a homodyne or heterodyne scheme is used (D2 and D3, respectively). Figure 1b,c show conventional frontends that ease the detector’s job by pre-amplifying or shifting the received radiation to lower frequencies. In a simple direct detector the antenna couples the incident radiation to a square-law detector or bolometer (Fig. 1a–D1) which determines the noise and thus usually needs to be cooled for high sensitivity, e.g.^12^. The front ends of classical radiometers, however, either incorporate a low noise amplifier (LNA)^6,13^ or a mixer, shown schematically in Fig. 1b,c, respectively. Amplification or downconversion of the incident THz electromagnetic waves to intermediate frequencies allows the use of standard room-temperature microwave detectors, transferring most of the noise budget to the frontend. These coherent techniques provide amplitude and phase of the received waves to a homodyne or heterodyne detector (D2 and D3, respectively in Fig. 1) leading to a fundamental noise penalty known as the quantum limit^14^. Cryogenic High Electron Mobility Transistor (HEMT)-based low noise amplifiers and Superconductor Insulator Superconductor (SIS) mixers are widely used as frontends of ultra-low noise instruments, achieving in some cases noise figures just few times higher than the quantum limit^15,16^.

Upconverting the radiation to optical frequencies (Fig. 1d) where room temperature photonic detectors are highly sensitive is a different approach proposed in^17–25^. In this approach, an optical local oscillator (laser pump) is modulated by the THz incident radiation in an efficient electro-optic modulator (EOM), thus upconverting the THz spectrum to optical sidebands. Detection is then performed with conventional photodetectors in a coherent or incoherent scheme. The noise and sensitivity performance of such a receiver should account for thermal noise of the modulator and its photon conversion efficiency, for both coherent and incoherent detection which will fundamentally result in different sensitivity limits.

The goal of this paper is to analyze the noise in electro-optic upconversion receivers, perform a fair comparison with traditional receivers (Fig. 1), and quantify the conditions under which upconversion results in better noise performance. The paper is organized as follows. Section 2 develops the noise analysis of an upconversion electro-optic receiver with direct, homodyne and heterodyne optical detection. Section 3 focuses on a comparison of upconverters with LNAs and mixers, starting from fundamental noise limits and continuing with noise limitations. Finally, we show quantitatively the conditions under which room-temperature upconversion THz receivers have the potential to exhibit sensitivity and noise comparable to those of cryogenic LNA and mixer-based receivers.

## Detection of thermal radiation via upconversion

In optical upconversion an optical pump (local oscillator) is modulated by millimeter-wave or THz thermal signals which appear as sidebands in the optical spectrum. Ideally, the modulation process is 100% efficient, i.e. all of the incoming THz photons are transformed into optical photons. Low photon conversion efficiencies *η*_*u*_ reduce the system sensitivity, as quantified in the remainder of the paper. The modulation can be a result of the sum-frequency-generation (SFG) and/or difference-frequency-generation (DFG) processes in an electro-optic modulator (EOM). However, the efficiency *η*_*u*_ achievable with off-the-shelf EOMs is too low for a sensitive receiver. The use of ultra high-Q resonant optics can significantly enhance the conversion efficiency of an EOM, reaching values (per mW pump power) on the order of 10^−2^ in X-band and 10^−5^ in W-band and as demonstrated in whispering-gallery mode (WGM) resonators^24–26^. These efficiency values are still orders of magnitude below the maximum fundamentally achievable by enhancing the spatial overlap between THz and optical modes with optimized mode-confining resonant structures^24,25,27^. Some thermal noise is coupled to the resonator due to its physical temperature, and is also upconverted along with the radiation collected by the antenna. Previous studies found that the equivalent temperature *T*_*e*_ of the upconverted noise due to the resonator’s thermal population can be below its physical temperature *T*_*p*_ as long as it is sufficiently overcoupled to the antenna^19,25^. As both the radiation collected by the antenna and the noise due to *T*_*e*_ are upconverted with the same efficiency *η*_*u*_, it may appear that the value of *η*_*u*_ is irrelevant for the upconverter’s noise figure as long as the photodetector is sensitive enough to low incoming powers. This is however not true since photon shot noise grows as *η*_*u*_ decreases.

**Figure 2:**
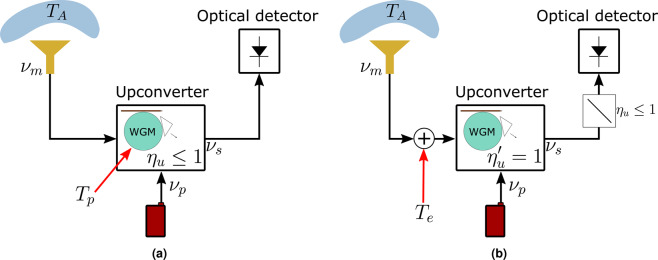
(**a**) Schematic of upconversion of THz radiation via electro-optic modulation in a high-Q WGM resonator. An optical pump at frequency *v*_*p*_ is coupled to the EOM WGM resonator through a prism, while the THz radiation (*v*_*m*_) is coupled from an antenna through, e.g. a dielectric waveguide as in^25^. The EOM is thermally populated due to its physical temperature *T*_*p*_ and has photon conversion efficiency *η*_*u*_. The modulated optical sideband (*v*_*S*_) is then coupled from the resonant modulator to a photodetector. (**b**) The upconverter can be modeled as an ideal unity-efficiency noiseless EOM, fed by a input-referred thermal source at equivalent temperature *T*_*e*_. The attenuator (beam splitter) at the output models the photon efficiency *η*_*u*_.

Figure 2a shows a non ideal upconverter coupled to an ideal antenna receiving THz thermal radiation centered at frequency *v*_*m*_ from a far field blackbody source at temperature *T*_*A*_. The upconverter is an efficient EOM that we depict as a high-Q WGM resonator pumped by a laser at frequency *v*_*p*_. Hence, optical sidebands are generated at frequencies *v*_*s*_ = *v*_*p*_ ± *v*_*m*_ due to SFG and/or DFG processes, and then photodetected. A SFG process is preferred since it is free of spontaneous parametric downconversion (SPDC) noise, and thus intrinsically noiseless^28–30^. We model this receiver by first making some simplifications. We consider all the coupled ambient noise as an equivalent input-referred source at temperature *T*_*e*_ as shown in Fig. 2b^25^. Then, the non-unity efficiency is modeled by a beamsplitter with coupling *η*_*u*_ before or after an ideal EOM^29,30^. This is because *η*_*u*_ is just the probability that one THz photon is upconverted instead of absorbed by the crystal resonator. After this, the EOM can be treated as 100% efficient and not thermally populated. Considering only a SFG process, photon statistics is identical at the input and output of such an ideal EOM. In general, the contributions of thermal and photon shot noise in the overall noise figure of the receiver differ for incoherent and coherent optical detection schemes, as discussed below.

### Direct detection

An incoherent detector responds to the instantaneous incident electromagnetic power. The complex representation of the THz electric field in a given polarization is given by

1 $$E(t,r)=A(t)\Phi ({\boldsymbol{r}})\exp (i2\pi {\nu }_{m}t)$$

where Φ(***r***) is the spatial field distribution, and *A*(*t*) = *a*_*r*_(*t*) + *ia*_*i*_(*t*) is a baseband complex amplitude normalized such that the instantaneous power incident on the EOM is calculated as $P(t)={|A(t)|}^{2}={a}_{r}^{2}(t)+{a}_{i}^{2}(t)$. The function Φ(***r***) is chosen for *A*(*t*) to have a square-root-of-power unit. *a*_*r*_(*t*) and *a*_*i*_(*t*) are real independent zero-mean Gaussian random processes with power spectral densities *S*(*ω*) = *S*_0_*H*(*ω*), where *S*_0_ is a constant and *H*(*ω*) is the EOM’s upconversion filter shape shifted to baseband and normalized such that *H*(0) = 1. We define the bandwidth of the upconverted radiation as the bandwidth of an equivalent rectangular filter delivering the same power:

2 $$\Delta \nu =\frac{1}{2\pi }{\int }_{-\infty }^{\infty }\,H(\omega )\,{\rm{d}}\omega .$$

This allows us to write

3 $$\langle {a}_{r}^{2}(t)\rangle =\langle {a}_{i}^{2}(t)\rangle =\frac{1}{2}\langle P(t)\rangle ={S}_{0}\Delta \nu ,$$

where the angle brackets denote statistical expected value, and ergodicity is assumed for all involved random processes. We assume the photodetector is able to track evenly the instantaneous fluctuations of the optical power, i.e., its bandwidth is much wider than Δ*v* and its frequency response is flat within *H*(*ω*). Thermal noise and dark currents in the photodetector are neglected, although they can later be included as additive uncorrelated noise.

**Figure 3:**
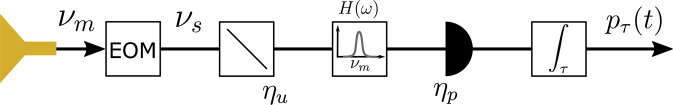
Schematic of a radiometer using an electro-optic modulator as an upconverter of THz radiation to the optical domain. After receiving and upconverting, incoherent (direct) detection is used.

An incoherent radiometer can therefore be built by upconverting the THz radiation and detecting it with the scheme shown in Fig. 3. Some laser pump can leak out from the EOM due to non perfect coupling to the WGM resonator. In order to detect only the upconversion sideband, such leaked pump must be filtered before the photodetector which might be a challenge especially for lower modulation (detection) THz frequencies. The beamsplitter with transmission coefficient *η*_*u*_ accounts for the upconverter’s photon efficiency, while the beamsplitter with transmission coefficient *η*_*p*_ models the photodetector quantum efficiency. The photocurrent is integrated since radiometry requires observing the scene during some interval $\tau \gg \Delta {\nu }^{-1}$ to get an estimate of the average received power with a given uncertainty. The output of the integrator *p*_*τ*_(*t*) is a sample mean over *τ* of the total radiometer input thermal power *P*(*t*) so that $\langle {p}_{\tau }\rangle =\langle P\rangle$ and *p*_*τ*_(*t*) is an estimation of the temperature of the observed scene, including antenna and ambient temperatures *T*_*A*_ and *T*_*e*_, respectively. Next, we determine the fluctuations (variance) of this measurement to quantify the radiometer sensitivity. Classically, excluding photon shot noise effects, this expression is the well-known radiometer equation^31^

4 $${\rm{var}}({p}_{\tau })=\frac{{\langle P\rangle }^{2}}{B\tau },$$

where *B* is the equivalent noise bandwidth^31,32^, $\langle P\rangle =\langle {P}_{A}\rangle +\langle {P}_{e}\rangle$, where $\langle {P}_{A}\rangle ={k}_{B}{T}_{A}\Delta \nu$ and $\langle {P}_{e}\rangle ={k}_{B}{T}_{e}\Delta \nu$, *k*_*B*_ is the Boltzmann constant, and temperatures are given in Rayleigh-Jeans units, i.e., temperature is power normalized by ${k}_{B}^{-1}\Delta {\nu }^{-1}$.

Equation 4 does not take into account photon shot noise, which is relevant at THz frequencies. A semi-classical derivation of the radiometer equation considers the fluctuation of the number of photons received by the detector during a time interval *τ*, which is determined by var(*p*_*τ*_). Mandel’s rule^33,34^ gives the probability *P*_*m*_ of detecting *m* photons within an interval *τ* with a photodetector with quantum efficiency *η*_*p*_:

5 $${P}_{m}=\langle \frac{{[{M}_{\tau }(t)]}^{m}}{m!}\exp [-{M}_{\tau }(t)]\rangle ,$$

where *M*_*τ*_(*τ*) is a random process quantifying the number of optical photons that would be *classically* detected during *τ*, and is equal to the number of incident photons reduced by *η* = *η*_*p*_*η*_*u*_. In terms of THz incident power, ${M}_{\tau }(t)=\frac{\eta \tau }{h{\nu }_{m}}{p}_{\tau }(t)$ where ${p}_{\tau }(t)=\frac{1}{\tau }{\int }_{t-\tau }^{t}\,P(t{\prime} )\,dt{\prime} =P(t)\ast f(t)$ is the power incident on the upconverter averaged during *τ*. It is equivalent to the convolution of *P*(*t*) with a function *f*(*t*) whose value is *τ*^−1^ for 0 ≤ *t* ≤ *τ* and 0 otherwise. Observe that after the upconverter the power is scaled by *G* = *η*_*u*_*v*_*s*_/*v*_*m*_, which can be greater than unity for a sufficiently efficient upconversion process. From the calculation of the second moment of Eq. (5), the variance in photon counts becomes

6 $${\rm{var}}(m)=\langle m\rangle +{\left(\frac{\eta \tau }{h{\nu }_{m}}\right)}^{2}{\rm{var}}[{p}_{\tau }(t)].$$

where ${\rm{var}}({p}_{\tau })=\langle {p}_{\tau }^{2}\rangle -{\langle P\rangle }^{2}$ since $\langle {p}_{\tau }\rangle =\langle P\rangle$. The second moment is now

7 $$\begin{array}{rcl}\langle {p}_{\tau }^{2}(t)\rangle  & = & \langle [{\int }_{-\infty }^{\infty }\,P(t-t{\prime} )f(t{\prime} )\,{\rm{d}}t{\prime} ][{\int }_{-\infty }^{\infty }\,P(t-t{\prime\prime} )f(t{\prime\prime} )\,{\rm{d}}t{\prime\prime} ]\rangle \\  & = & {\int }_{-\infty }^{\infty }\,{\int }_{-\infty }^{\infty }\,\mathop{\underbrace{\langle P(t-t{\prime} )P(t-t{\prime\prime} )\rangle }}\limits_{{R}_{PP}(t{\prime} -t{\prime\prime} )}f(t{\prime} )f(t{\prime\prime} )\,{\rm{d}}t{\prime} \,{\rm{d}}t{\prime\prime} \\  & = & \frac{1}{2\pi }{\int }_{-\infty }^{\infty }\,{S}_{PP}(\omega ){|F(\omega )|}^{2}\,{\rm{d}}\omega ,\end{array}$$

where ${|F(\omega )|}^{2}=\frac{{\sin }^{2}(\omega \tau \,/\,\mathrm{2)}}{{(\omega \tau /\mathrm{2)}}^{2}}$ is the square of the Fourier transform of *f*(*t*), *R*_*PP*_(*t*′ − *t*″) is the autocorrelation function of *P*(*t*) and we used the fact it is equal to the inverse Fourier transform of the power spectral density of *P*, denoted as *S*_*PP*_(*ω*). The autocorrelation function is given by

8 $${R}_{PP}(\delta t)=\langle P(t)P(t+\delta t)\rangle =\langle {a}_{r}^{2}(t){a}_{r}^{2}(t+\delta t)\rangle +2\langle {a}_{r}^{2}(t){a}_{i}^{2}(t+\delta t)\rangle +\langle {a}_{i}^{2}(t){a}_{i}^{2}(t+\delta t)\rangle .$$

For correlated Gaussian processes *x* and *y*, $\langle {(xy)}^{2}\rangle ={\rm{var}}(x){\rm{var}}(y)+2{\langle xy\rangle }^{2}$^35^, and Eq. (8) can be rewritten as

9 $${R}_{PP}(\delta t)={\langle P\rangle }^{2}+2{\langle {a}_{r}(t){a}_{r}(t+\delta t)\rangle }^{2}+2{\langle {a}_{i}(t){a}_{i}(t+\delta t)\rangle }^{2},$$

and its Fourier transform is

10 $${S}_{PP}(\omega )=2\pi {\langle P\rangle }^{2}\delta (\omega )+4{ {\mathcal F} }_{(\delta t)}\{{R}_{aa}^{2}(\delta t)\},$$

where ${R}_{aa}(\delta t)=\langle {a}_{r}(t){a}_{r}(t+\delta t)\rangle =\langle {a}_{i}(t){a}_{i}(t+\delta t)\rangle$ is the autocorrelation of *a*_*r*_(*t*) and *a*_*i*_(*t*) whose Fourier pair is the power spectral density *S*(*ω*). Thus we can write,

11 $${S}_{PP}(\omega )=2\pi {\langle P\rangle }^{2}\delta (\omega )+\frac{4}{2\pi }S(\omega )\ast S(\omega ).$$

Since in radiometry $\tau \gg \Delta {\nu }^{-1}$, the bandwidth of *F*(*ω*) is much narrower than that of *S*_*PP*_(*ω*). Inserting (11) in (7) we find the variance as

12 $${\rm{var}}[{p}_{\tau }(t)]\simeq \frac{4}{2\pi \tau }{S(\omega )\ast S(\omega )|}_{\omega =0}=\frac{{\langle P(t)\rangle }^{2}}{B\tau },$$

where *F*(0) = 1, $$\frac{1}{2\pi }{\int }_{-\infty }^{\infty }\,{|F(\omega )|}^{2}\,d\omega =\frac{1}{\tau }$$, and

13 $$B=\frac{1}{2\pi }\frac{{({\int }_{-\infty }^{\infty }H(\omega ){\rm{d}}\omega )}^{2}}{{\int }_{-\infty }^{\infty }\,{H}^{2}(\omega )\,{\rm{d}}\omega }$$

is not surprisingly the noise equivalent bandwidth defined in^31^ for classical radiometry. In Eq. (12) we used Eqs. (2) and (3) to write ${S(\omega )\ast S(\omega )|}_{\omega =0}={S}_{0}^{2}{\int }_{-\infty }^{\infty }\,{H}^{2}(\omega )\,{\rm{d}}\omega$, knowing that ${S}_{0}{\int }_{-\infty }^{\infty }\,H(\omega )\,{\rm{d}}\omega =\pi \langle P\rangle$. Inserting (12) into (6) yields ${\rm{var}}(m)=\langle m\rangle +{\langle m\rangle }^{2}/(B\tau )$, or equivalently in terms of measured power,

14 $${\rm{v}}{\rm{a}}{\rm{r}}({p}_{\tau }(t))=\frac{{(\langle {P}_{A}\rangle +\langle {P}_{e}\rangle )}^{2}}{B\tau }\left(1,+,\frac{h{\nu }_{m}B}{\eta (\langle {P}_{A}\rangle +\langle {P}_{e}\rangle )}\right).$$

Since the estimation of power received by the antenna is the output of the radiometer without an ambient noise offset, ${P}_{A}={p}_{\tau }(t)-\langle {P}_{e}\rangle$, then (14) represents the noise in the measurement and is thus a semi-classical radiometer equation. The difference between (14) and the classical radiometer equation of (4) is a photon shot noise factor which is negligible only when $h{\nu }_{m}\ll \eta {k}_{B}({T}_{A}+{T}_{e})$. This quantum noise term is however not negligible in the THz region. For example, even for an optimistic value of 10% photon conversion efficiency at 300 GHz, the classical radiometer equation (14) underestimates the measurement uncertainty (standard deviation) by about 18% at room temperature. For *η* = 1, (14) represents the minimum uncertainty achievable by any detector when measuring power from a thermal source^36^.

### Homodyne detection

**Figure 4:**
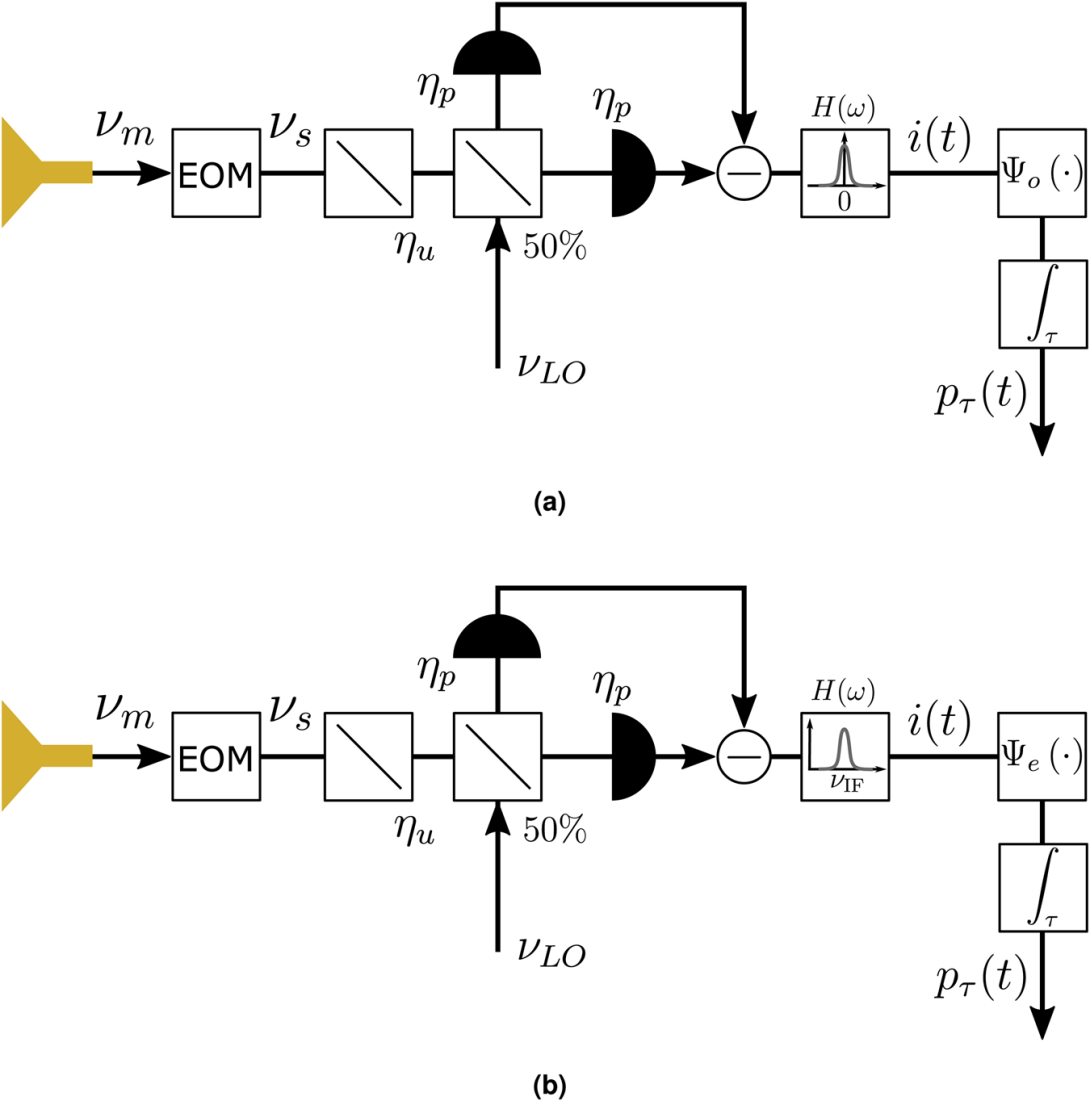
Schematic of a radiometer using an electro-optic modulator as an upconverter of THz radiation to the optical domain, followed by (**a**) homodyne or (**b**) heterodyne coherent detection. In each of these schemes, the fraction of laser pump *v*_*p*_ potentially leaking out of the EOM due to imperfect coupling to the WGM resonator does no need to be filtered before photodetection. This is because the pump-induced photocurrent lies at an intermediate frequency close to *v*_*m*_ which is in a real scenario very far from the response bandwidth of the photodetector.

In homodyne detection, the incoming radiation at *v*_*S*_ is superimposed with a strong local oscillator (LO) at the same frequency *v*_LO_ = *v*_*S*_, and the generated beatnote measured with a balanced photodetector to reduce the effect of LO fluctuations (Fig. 4a). The detected signal is the electric field component in phase with the LO. Assuming ideal photodetectors with quantum efficiency *η*_*p*_ < 1, this detection is associated with noise of a fundamental nature, analogous to the photon shot noise in direct detection discussed in the previous section. The noise is manifested as a photocurrent with zero-mean Gaussian statistics and a white spectrum. In order to filter equally both signal and noise, we restrict our attention to the photocurrent whose spectrum lies within the filter shape *H*(*ω*) and assume the incoming radiation is flat within Δ*v*, i.e. the received spectrum is determined by a post-processing filter.

A formal quantum analysis of a homodyne detector observing an arbitrary electromagnetic quantum state attributes the noise to three independent sources: the vacuum fluctuations in the signal and in each detector^37,38^. Interestingly, with this interpretation, the total noise matches that resulting from a classical analysis of electron shot noise in the photodetectors as long as the local oscillator is not in a non-classical (squeezed) state. Regardless of the interpretation, the filtered photocurrent difference (in electrons per second) at the output of a homodyne detector is given by^37^:

15 $$i(t)=\sqrt{\frac{{\eta }_{p}{P}_{{\rm{LO}}}}{h{\nu }_{s}}}n(t)+\frac{2{\eta }_{p}}{h{\nu }_{s}}\sqrt{{P}_{{\rm{LO}}}}Re\left[\sqrt{{\eta }_{u}\frac{{\nu }_{s}}{{\nu }_{m}}}A(t)\right]$$

where *n*(*t*) is a baseband zero-mean Gaussian process with power spectral density *H*(*ω*) and mean power $\langle {n}^{2}(t)\rangle =\Delta \nu$, *η*_*u*_ is the EOM photon conversion efficiency and *η*_*p*_ is the quantum efficiency of the photodetectors such that *η *=* η*_*p*_*η*_*u*_. We can rewrite (15) as

16 $$i(t)={C}_{n}n(t)+{C}_{s}\mathrm{Re}[A(t)],$$

where ${C}_{n}={G}^{-1/2}\sqrt{\frac{\eta {P}_{{\rm{LO}}}}{h{\nu }_{m}}}$, ${C}_{s}=\frac{2\eta }{h{\nu }_{m}}{G}^{-1/2}\sqrt{{P}_{{\rm{LO}}}}$ and *P*_LO_ is the LO power. The zero-mean Gaussian processes *n*(*t*) and *Re*[*A*(*t*)] = *a*_*r*_(*t*) are independent and hence their superposition is a zero-mean Gaussian process with added variances. Therefore, we can write

17 $$i(t)={\left({C}_{n}^{2}+\frac{\langle P\rangle }{2\Delta \nu }{C}_{s}^{2}\right)}^{1/2}{n}_{t}(t),$$

where *n*_*t*_(*t*) is another zero-mean Gaussian process with power spectral density *H*(*ω*) such that $\langle {n}_{t}^{2}(t)\rangle =\Delta \nu$. In order to retrieve the power received by the antenna from the measured *i*(*t*), a postprocessing step is required via a transformation Ψ_*o*_[*i*(*t*)] that maps the photocurrent into an estimation of the incoming power. To that end we define

18 $$p(t)={\Psi }_{o}[i(t)]=\frac{2}{{C}_{s}^{2}}{i}^{2}(t)-2\Delta \nu \frac{{C}_{n}^{2}}{{C}_{s}^{2}}$$

as the estimator of the received power since $\langle p(t)\rangle =\langle P\rangle$, so the uncertainty in the measurement is given by the variance of *p*(*t*) once integrated during *τ*. The output after integration is *p*_*τ*_(*t*) = *p*(*t*)*f(*t*), with variance given by (7). The autocorrelation of *p*(*t*) is

19 $${R}_{pp}(\delta t)=\langle p(t)p(t+\delta t)\rangle ={\langle P\rangle }^{2}+2{\left(\frac{h{\nu }_{m}}{2\eta }+\frac{\langle P\rangle }{\Delta \nu }\right)}^{2}{\langle {n}_{t}(t){n}_{t}(t+\delta t)\rangle }^{2}.$$

where we replaced $\frac{{C}_{n}^{2}}{{C}_{s}^{2}}=\frac{h{\nu }_{m}}{4\eta }$. Taking the Fourier transform of (19) to get the power spectral density of *p*(*t*) and then inserting it in (7), assuming $\tau \gg \Delta {\nu }^{-1}$ and following a similar procedure as in Section 2.1, we obtain

20 $${\rm{var}}({p}_{\tau }(t))=\frac{2}{B\tau }{\left(\frac{h{\nu }_{m}\varDelta \nu }{2\eta }+\langle {P}_{A}\rangle +\langle {P}_{e}\rangle \right)}^{2}.$$

Equation (20) also characterizes the fluctuations in the estimation of the mean power received by the antenna, and is a radiometer equation for a homodyne receiver. This result can be interpreted as twice noisier than that obtained from the classical radiometer equation, provided that an additional thermal source of quantum origin with Rayleigh-Jeans temperature of *hv*_*m*_/(2*k*_*B*_*η*) is included, as described by the first term in parenthesis in (20). Such an additional noise source reaches a temperature of about 72 K for an (optimistic) photon-efficiency of *η* = 0.1 at 300 GHz, which might be already significant for high sensitivity applications. In coherent detection, photon shot noise appears as fluctuations in the field in contrast to incoherent detection, where photon shot noise produces fluctuations in power. As a consequence, the radiometric sensitivity drops faster with the photon shot noise term *hv*_*m*_/*η* in coherent detection than in incoherent. The difference becomes significant for higher frequencies and/or lower efficiencies. In the low frequency limit $h{\nu }_{m}/({k}_{B}\eta )\ll ({T}_{e}+{T}_{A})$, Eq. (19) converges to the second moment of the square of a zero-mean Gaussian process *x*, $\langle {x}^{4}\rangle =3{\rm{var}}{(x)}^{2}$^35^. Hence, in this limit (20) corresponds to the classical radiometer equation expected when observing only a single component (in-phase or quadrature) of the incoming thermal radiation.

### Heterodyne detection

Heterodyne detection can be seen as a generalization of the homodyne scheme, when the local oscillator is at frequency *v*_LO_ = *v*_*S*_ + *v*_IF_ where *v*_IF_ > Δ*v*/2 is an intermediate frequency within the photodetector bandwidth (see Fig. 4b). As in the homodyne case, we consider the received spectrum to be determined by a post-processing filter. Formally, noise in heterodyne detection is attributed to vacuum fluctuations in the signal and image spectra, as well as in the detectors^37^. All sources are independent so they can be combined, and the resulting filtered heterodyne photocurrent difference is^37^

21 $$i(t)={C}_{n}{n}_{{\rm{shot}}}(t)\ast {h}_{{\rm{IF}}}(t)+{C}_{s}Re[A(t)\exp (i2\pi {\nu }_{{\rm{IF}}}t)]$$

where *n*_shot_(*t*) is a zero-mean Gaussian process with white spectrum and unity power spectral density, and *h*_IF_(*t*) is the impulse response of the filter shape *H*_IF_(*ω*), the same as *H*(*ω*) in (2) but centered at *v*_IF_. Since *v*_IF_ > Δ*v*/2, the convolution ${n}_{{\rm{shot}}}(t)\ast {h}_{{\rm{IF}}}(t)={\rm{R}}{\rm{e}}[({n}_{r}(t)+i{n}_{i}(t))\exp (i2\pi {\nu }_{{\rm{IF}}})]$, so we can rewrite (21) as

22 $$i(t)=[{C}_{n}{n}_{r}(t)+{C}_{s}{a}_{r}(t)]\cos \,\mathrm{(2}\pi {\nu }_{{\rm{IF}}}t)+[{C}_{n}{n}_{i}(t)+{C}_{s}{a}_{i}(t)]\sin \,\mathrm{(2}\pi {\nu }_{{\rm{IF}}}t)$$

where *n*_*r*_(*t*) and *n*_*i*_(*t*) are baseband zero-mean independent Gaussian processes with power spectral densities *H*(*ω*), and mean power Δ*v*. We define an estimator *p*(*t*) = Ψ_*e*_[*i*(*t*)] with a mean $\langle P\rangle$ that computes the sum of squares of the in-phase and quadrature components of the current:

23 $$\begin{array}{rcl}p(t) & = & \frac{4}{{C}_{s}^{2}}[{({\nu }_{{\rm{IF}}}{\int }_{t-{\nu }_{{\rm{IF}}}^{-1}}^{t}i(t)\cos \mathrm{(2}\pi {\nu }_{{\rm{IF}}}t){\rm{d}}t)}^{2}+{({\nu }_{{\rm{IF}}}{\int }_{t-{\nu }_{{\rm{IF}}}^{-1}}^{t}i(t)\sin \mathrm{(2}\pi {\nu }_{{\rm{IF}}}t){\rm{d}}t)}^{2}]-2\frac{{C}_{n}^{2}}{{C}_{s}^{2}}\Delta \nu \\  & = & \frac{4}{{C}_{s}^{2}}\left[{\left(\frac{1}{2}{C}_{{\rm{eq}}}{n}_{1}(t)\right)}^{2}+{\left(\frac{1}{2}{C}_{{\rm{eq}}}{n}_{2}(t)\right)}^{2}\right]-2\frac{{C}_{n}^{2}}{{C}_{s}^{2}}\Delta \nu \end{array}$$

where *n*_1_(*t*) and *n*_2_(*t*) are random processes with the same statistics as *n*_*r*_(*t*), and we use ${C}_{{\rm{eq}}}^{2}={C}_{s}^{2}\frac{\langle P\rangle }{2\Delta \nu }+{C}_{n}^{2}$ because the processes in (22) are all statistically independent. After integration, we have *p*_*τ*_(*t*) = *p*(*t*)*f(*t*) as before, and the autocorrelation becomes

24 $$\begin{array}{rcl}{R}_{pp}(\delta t) & = & {\langle P\rangle }^{2}+{\left(\frac{{C}_{{\rm{eq}}}^{2}}{{C}_{s}^{2}}\right)}^{2}[\langle ({n}_{1}^{2}(t)+{n}_{2}^{2}(t))({n}_{1}^{2}(t+\delta t)+{n}_{2}^{2}(t+\delta t))\rangle -4\Delta {\nu }^{2}]\\  & = & {\langle P\rangle }^{2}+4{\left(\frac{{C}_{{\rm{eq}}}^{2}}{{C}_{s}^{2}}\right)}^{2}{R}_{nn}^{2}(\delta t),\end{array}$$

where ${R}_{nn}(\delta t)=\langle {n}_{j}(t){n}_{j}(t+\delta t)\rangle$ for *j* = 1, 2. Following a similar procedure as in Section 2.1 and inserting the result into Eq. (7), we obtain

25 $${\rm{var}}({p}_{\tau }(t))=\frac{1}{B\tau }{\left(\frac{h{\nu }_{m}\Delta \nu }{2\eta }+\langle {P}_{A}\rangle +\langle {P}_{e}\rangle \right)}^{2}\mathrm{}.$$

Therefore, the same conclusions may be derived when comparing both homodyne or heterodyne detection with direct detection. For radiometry, however, the sensitivity of heterodyne detection is two times better than that of homodyne detection since both in-phase and quadrature components are observed. Thus, (25) becomes the classical radiometer Eq. (4) in the low frequency limit.

Without post-integration ($\tau \ll \Delta {\nu }^{-1}$), we can obtain the noise floor of the coherent THz receiver by taking the root-mean-square power (standard deviation) of homodyne or heterodyne photocurrents (Eqs. (17) or (22) repectively) and dividing by the overall receiver gain, i.e., the ratio between the output photocurrent power and the input THz power. Such noise floor is the usual figure of merit employed to characterize the sensitivity of the receiver while observing a monochromatic THz signal^39,40^. From (17) and (22) we obtain a noise floor whose power spectral density has the form 2*k*_*B*_(*T*_*A*_ + *T*_*e*_) + *hv*_*m*_/*η*. The minimum of such noise floor agrees with the highest possible detection sensitivity reported previously^19^. However our result is more general than previous estimations^39,40^ as it depends only on the effective thermal noise coupled to the upconverter and its photon conversion efficiency. Indeed, the quantum-limited sensitivity term *hv*_*m*_/*η* was neglected in previous studies^39,40^ and only the photodetector-induced shot noise was considered. The latter is not fundamental as can be made arbitrarily small in the strong pump limit^19,37^, which is the consideration we made above for the optical local oscillator at *v*_LO_.

Heterodyne detection relies on the downconversion to an intermediate frequency *v*_IF_, which is the difference between local oscillator frequency *v*_LO_ and that of the signal *v*_*m*_ = *v*_LO_ + *v*_IF_. However, the image centered at *v*_LO_ − *v*_IF_ is also downconverted. Hence, normally the superposition of both, signal and image bands are detected leading to a double sideband mixer (DSB). This can be avoided by using a two sidebands mixer (2SB)^41^ which retrieves both bands separately. Alternatively, the image band can also be filtered, but this is not trivial in ultra-low noise receivers such as the ones found in radio astronomy applications, since band-rejection filters and backshorts might require cooling in order to reduce their thermal noise contribution^41^. Optical heterodyne detection after upconversion is analogous to a single-sideband architecture (based on e.g. SIS mixers) with no requirement for cooled filters or backshorts for sideband rejection. In this case, single-sideband detection is guaranteed since radiation in the image sideband is not existent (except for its vacuum fluctuations) in the optical domain.

## Comparison with low noise amplifiers and mixers

In the previous section the uncertainty in the radiometric measurement was quantified when thermal radiation is upconverted and then detected incoherently or coherently in either homodyne or heterodyne schemes. It was shown that on one hand both coherent detection schemes introduce white noise of quantum origin which is statistically independent from thermal noise and ideally has a power spectral density of half a photon per unit time per unit bandwidth. On the other hand, incoherent detection introduces power fluctuations of quantum origin, which increases the uncertainty in the power measurement. In this section, we derive an equivalent noise temperature for coherent and incoherent upconversion radiometry and compare with conventional receivers based on low noise amplifiers and mixers in terms of fundamental limits. Further comparisons are done between state-of-the-art conventional receivers and efficient upconverters.

### Fundamental limits

The upconverter noise study discussed in previous sections and its conclusions are also valid for a receiver with no upconversion stage, but instead consisting of a THz mixer that downconverts the radiation to an intermediate frequency, allowing heterodyne detection. For that we just need to set ${\nu }_{s}={\nu }_{m}$ and account for losses by *η*. Therefore, conventional downconversion radiometers (based on e.g., SIS mixers) exhibit a minimum noise temperature (in Rayleigh-Jeans units) known as the quantum limit *T*_*q*_ = *hv*_*m*_/(2*k*_*B*_). The same fundamental limit exists with the upconversion stage and coherent detection. This can be regarded to be the penalty incurred for the simultaneous knowledge of both amplitude and phase of the incoming radiation, and not a consequence of the upconversion process. Interestingly, it was recently suggested^42^ that the quantum limit can be overcome when cross correlation between two heterodyne detectors observing the same source is performed. This is the typical situation appearing in interferometry radio astronomy. Heisenberg’s uncertainty principle is not violated in this case since a relative and not absolute phase measurement is done with an uncertainty below the quantum limit. If experimentally verified further, this technique has potential for ultra-low noise interferometry radio astronomy via upconversion and optical cross-correlation as suggested in^25^.

Amplification of the incoming radiation also leaves amplitude and phase available for easy detection, so the same penalty must be incurred. Indeed, it can be shown that fundamentally any phase-insensitive amplification process is subject to a minimum noise temperature of *T*_*q*_(1 − 1/*G*) yielding the same quantum limit in the high-gain limit^14,43,44^. The study in Section 2 is general enough to be applicable for an amplification stage instead of an upconverter, followed by coherent or incoherent detection schemes. For this we set *v*_*S*_ = *v*_*m*_ and replace *η* = *G* by the amplifier’s effective gain, accounting for the losses as well. The amplifier’s total noise temperature referred to the input *T*_*e*_ as measured with the conventional Y-factor method, includes the quantum limit as well as the thermal noise due to its physical temperature. Hence, even though in the high-gain limit as $G={\eta }_{u}\approx \eta \to \infty$, the photon shot noise term in Eqs. (14), (20) and (25) vanishes, both direct and heterodyne detection after large amplification yield the same quantum-limited radiometric sensitivity because *T*_*e*_ ≥ *T*_*q*_ already includes the quantum limit temperature. In short, we can highlight two main points:

- Radiometers based on LNAs, mixers (downconverters) and upconverters followed by heterodyne optical detection all have a sensitivity described by Eq. (25) and are quantum-limited by *T*_*q*_. Homodyne detection after upconversion is half as sensitive as heterodyne in terms of the variance.
- Radiometers based on upconverters followed by a direct optical detection stage, do not incur the fundamental minimum noise of temperature *T*_*q*_ although photon shot noise is present as described by Eq. (14). Notice that in direct detection, photon shot noise, and therefore the measurement uncertainty, is input-dependent, i.e., depends on *T*_*A*_ and *T*_*e*_.

### Equivalent noise temperature of the upconverter

The main advantage of upconversion via WGM resonators is that the input-referred thermal contribution *T*_*e*_ can be considerably lower than the physical temperature of the resonator. Such low noise temperatures would dramatically outperform those of conventional receivers (LNAs or mixers) in the THz region at room temperature. However, as discussed before, the classical radiometer equation of (4) does not apply since photon shot noise can mostly determine the radiometric uncertainty leading to actual noise temperatures way above *T*_*e*_. In order to quantify this, we rewrite the uncertainty in the radiometric measurement given in (14), (20) and (25) in terms of temperature uncertainty of the observed scene var(*T*). Then, we define the additive noise temperature *T*_*n*_ as that which should be added to *T*_*A*_ + *T*_*e*_ in order to obtain the actual measurement uncertainty var(*T*) or var(*p*_*τ*_) while using the classical radiometer equation. This is a figure of merit that allows us to compare with conventional LNAs and mixers where using (4) is legitimate. For heterodyne detection, we obtain the time-bandwidth-normalized uncertainty (standard deviation)

26 $$\sigma =\sqrt{{\rm{var}}({\rm{T}})B\tau }=\frac{h{\nu }_{m}}{2{k}_{B}\eta }+{T}_{A}+{T}_{e},$$

from which the effective noise *σ* − *T*_*A*_ − *T*_*e*_ temperature is

27 $${T}_{n}=\frac{{T}_{q}}{\eta }.$$

The result of (27) is simple and independent from *T*_*A*_ because (25) is obtained from (4) by just adding temperature terms. This is not the case with direct detection where

28 $$\sigma =({T}_{A}+{T}_{e})\sqrt{1+2\left(\frac{B}{\Delta \nu }\right)\left(\frac{{T}_{q}}{\eta }\right)\left(\frac{1}{{T}_{A}+{T}_{e}}\right)},$$

from which

29 $${T}_{n}=({T}_{A}+{T}_{e})\left[\sqrt{1+2\left(\frac{B}{\Delta \nu }\right)\left(\frac{{T}_{q}}{\eta }\right)\left(\frac{1}{{T}_{A}+{T}_{e}}\right)}-1\right].$$

The ratio *B*/Δ*v* depends on the filter shape and is on the order of unity. The additive noise temperature described by (29) is input dependent since (14) can not be written in the form of (4) by simply adding independent noise temperatures. This means that the photon shot noise added by direct detection depends on the temperature of the scene observed *T*_*A*_. As a general rule, heterodyne detection is better in terms of noise than direct detection when the number of detected photons is sufficiently high. This can be due to either low frequencies, high efficiencies, high observation temperatures or a combination of them.

**Figure 5:**
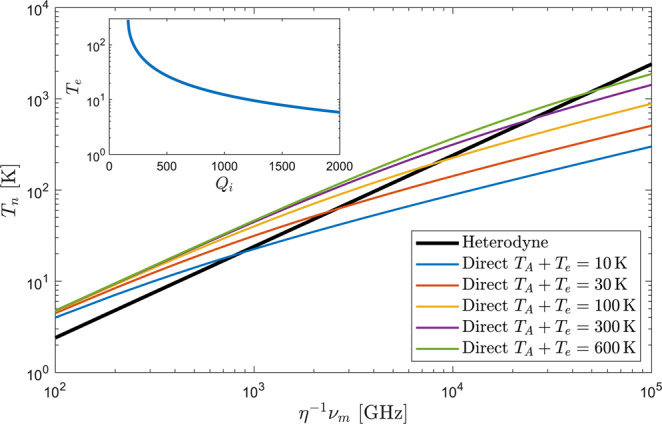
Additive noise temperature *T*_*n*_ of an upconverter followed by direct and heterodyne optical detection schemes as a function of the THz detection frequency normalized by the efficiency. Direct detection noise is plotted for different input temperatures. In the inset the upconverter’s effective noise temperature *T*_*e*_ for a WGM resonator working at room-temperature (290 K) as a function of the THz intrinsic quality factor. It is assumed the resonator is excited with a THz mode of azimuthal number *m*_*ϕ*_ = 4, and overcoupled to the antenna such that the intra-cavity power is about 6 times larger than the incoming power, as realized experimentally in^25^.

To determine the conditions under which incoherent detection is better than heterodyne, we plot for either scheme the values of *T*_*n*_ as a function of the detection frequency normalized by the efficiency *v*_*m*_/*η* as shown in Fig. 5. We assumed a Lorentzian filter shape for which *B*/Δ*v* = 2. Direct detection curves are generated for several input temperatures showing always a lower slope than the heterodyne curve, thus having an intersection point. A given WGM upconverter observing a scene with temperature *T*_*A*_ at frequency *v*_*m*_ has a fixed value of *T*_*e*_ which is determined by the THz intrinsic quality factor *Q*_*i*_ of the resonator^25^ as depicted in the inset of Fig. 5. Let us assume as an example, that at this point for a given value of *η*, heterodyne detection achieves lower noise than incoherent detection. Then, for lower efficiencies *η* we move to the right in the horizontal axis of Fig. 5 and after the intersection point direct detection yields better noise performance. The same argument can be followed by fixing instead the efficiency while increasing the detection frequency. The same behavior is observed for any input temperature, but the intersection point occurs for larger *v*_*m*_/*η* the hotter the input. After the intersection point it is thus preferred to photon-count the output of the upconverter, also because in this case the noise is not fundamentally lower-bounded by *T*_*q*_. Notice that for *η* = 1, the heterodyne detection curve represents the standard quantum limit. The final radiometric uncertainty is obtained from the classical radiometer equation considering as overall system temperature *T*_*A*_ + *T*_*e*_ + *T*_*n*_.

### Noise estimations

Conventionally, noise in radiometers is characterized by the so-called Y-factor method, where the mean power at the radiometer’s output is measured while observing cold and hot input thermal sources. The power offset is attributed to the additive noise contribution of the radiometer. Then, the classical radiometer equation of (4) is used to estimate the variance in the power measurement. This method is accurate for amplification, downconversion and coherent detection after upconversion since in all cases photon shot noise is manifested as actual exchangeable power. However, as discussed earlier, Y-factor measurements would underestimate the noise in radiometers based on low-efficiency upconversion followed by a direct detection scheme. For this reason, the radiometric variability *σ* obtained from the measurement variance is the appropriate figure of merit to compare the different detection approaches. From it, the noise additive temperature *T*_*n*_ defined in the previous section can be calculated.

Absorption losses in nonlinear crystals such as lithium niobate grow with frequency which negatively affects the efficiency with an approximately linear frequency dependence in the THz region. On the other hand, shorter THz wavelengths naturally enhance the spatial overlap between optical and THz modes making the process more efficient, although harder to phase-match. *T*_*e*_ also increases naturally with losses due to the reduced THz intrinsic Q factor^25^. This can be compensated by coupling more strongly to the resonant THz mode, at the expense of decreasing the intra-cavity power enhancement, and thus, the efficiency. Hence, the design of the WGM resonator geometry and coupling mechanism must be optimized for a given frequency since losses, coupling and phase-matching conditions are strongly frequency dependent. For simplicity, in this section, the noise estimation is done as a function of the frequency while considering fixed *T*_*e*_ and *η*.

**Figure 6:**
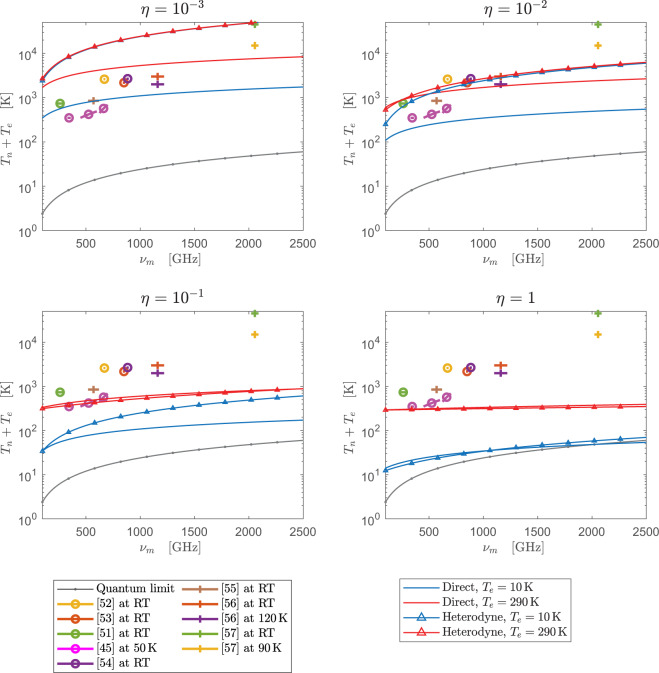
Noise introduced by the upconverter followed by direct and heterodyne optical detection schemes. The calculations are done for different *η* and *T*_*e*_ values and the observation of a scene at *T*_*A*_ = 2.7 K is assumed. A comparison is done with state-of-the-art LNAs and mixers^45,51–57^.

Figure 6 shows the overall noise temperature *T*_*e*_ + *T*_*n*_ introduced by an upconverter followed by direct and heterodyne optical detection schemes respectively. Observation of a cold source at 2.7 K is assumed while a Lorentzian filter shape is considered for the calculations in the incoherent case. The results are plotted for four efficiency values ranging from from *η* = 10^−3^ to *η* = 1 and two effective thermal noise temperatures: *T*_*e*_ = 10 K and *T*_*e*_ = 290 K. The inset of Fig. 5 shows that *T*_*e*_ ≈ 10 K can be realized in a WGM resonator with *Q*_*i*_ ≈ 1200 working at room temperature (290 K and overcoupled such that the intra-cavity power enhancement factor is on the order of the one realized experimentally in^25^. On the other hand, *T*_*e*_ = 290 K can be accomplished with resonators whose intrinsic quality factors are as low as 100.

As expected, direct detection is significantly better than heterodyne as the efficiency decreases. Indeed, for coherent detection with efficiencies as low as *η* = 10^−3^ the photon shot noise contribution greatly surpasses classical thermal fluctuations, producing similar curves for both values of *T*_*e*_. Moreover, since direct detection does not incur a fundamental noise penalty as coherent detection does, the radiometer noise is below the quantum limit at sufficiently high frequencies for unity efficiency and *T*_*e*_ = 10 K. This phenomena cannot happen if detection after the upconverter is coherent. A comparison is done between the predicted results for the upconverters and some state-of-the-art millimeter and submillimeter wave LNAs and mixers reported in the literature. It can be seen how an efficient room-temperature electro-optic upconverter followed by a direct optical detection stage may be competitive with conventional (even cryogenic) LNA’s and mixers in the THz range. For the sensitivity improvement with respect to the compared room-temperature devices to be significant, efficiencies above 0.1% and 1% must be accomplished for *T*_*e*_ = 10 K and *T*_*e*_ = 290 K respectively. A ten-fold increase in efficiency in both cases, would result in comparable or better sensitivity than HEMT LNAs cooled to 50 K^45^. In contrast, a heterodyne detection scheme requires efficiency values way above 1% to be competitive with conventional receivers. In this sense, an efficiency on the order of 10% would make room-temperature coherent detection after upconversion competitive with the cooled LNAs of^45^. Nevertheless, the benefits of upconversion are more evident at frequencies above 1 THz where noise in LNAs and mixers grows faster than in the upconverter.

## Conclusion

In this paper, we present a noise analysis of THz detectors and show the potential of high-Q THz-to-optical electro-optic modulation for high sensitivity detection of thermal radiation. The sum-frequency-generation (SFG) upconversion (Fig. 1d) is an alternative frontend to conventional receivers i.e., low noise amplifiers (LNAs) (Fig. 1b) and downconverters (mixers) (Fig. 1c). This type of detector can serve as a room-temperature THz photon counter, since photons are indirectly counted in the optical domain in contrast to direct THz photon counters which must be cooled (e.g., cryogenic THz bolometers).

The above conclusion is a result of an analysis of fundamental limits arising when detecting the THz-modulated light with direct, homodyne and heterodyne schemes. A comparison is done with the quantum limit widely used in radiometers based on LNAs and mixers. We conclude that a SFG upconverter does not fundamentally introduce noise, in contrast to an amplifier or downconverter. Therefore, the same quantum limit found with amplification or downconvertion arises only when optical detection is done coherently with a homodyne or heterodyne scheme. On the other hand, when optical detection is done incoherently (e.g., with an optical photon counter), no additional noise is present at the conventional quantum limit but photon shot noise still produces intensity fluctuations that are inversely proportional to the upconverter’s photon conversion efficiency, as shown in Eq. (14).

In addition to fundamental limits, we derive analytical expressions for the radiometric uncertainty in a SFG upconversion receiver. It is found that such a radiometer could be characterized with the conventional Y-factor method only when coherent (homodyne or heterodyne) optical detection is performed. In this case, the classical radiometer equation can be applied as long as a thermal source of quantum origin at temperature *T*_*q*_/*η* is added to all remaining thermal noise contributions of the system. When incoherent optical detection is performed instead, Y-factor measurements would significantly underestimate the noise of the upconversion radiometer. Moreover, the classical radiometer equation is not valid in this case. However, in an intent to compare both detection approaches, an equivalent noise temperature of quantum origin is defined. When added to the remaining thermal contributions of the system, this allows the use of the radiometer equation. Not surprisingly, such an equivalent temperature depends on the other thermal sources in the system, due to the “input dependance” of the photon count variance as noted by Haus^44^.

Finally, a noise comparison between state-of-the-art LNAs and mixers and the theoretical predictions in upconverters are presented. It is shown that upconverter-based THz radiometers can greatly surpass conventional ones in terms of sensitivity, provided that photon conversion efficiencies above 1% are accomplished. This requirement can be relaxed down to 0.1% if WGM resonators with intrinsic THz quality factors above 1000 are available. So far, WGM-based EOM’s have demonstrated photon conversion efficiencies per mW of optical pump power on the order of 0.0025% and 0.1% at room temperature, and 3% in a cryostat^24–26^. Further optimizations in the geometry of the resonator and the optical excitation are possible to increase the overlap between optical and THz modes, thus enhancing efficiency. Moreover, other nonlinear materials can be explored because of their trade-off between second-order susceptibility and intrinsic quality factors in both optical and THz domains. It is worth mentioning that other upconversion approaches^46–50^ are promising due to the potentially high photon conversion efficiencies.

It should be noted that, due to the high-Q optical modes, upconversion via electro-optic modulation in whispering-gallery mode resonators is inherently narrowband. The optical resonances could be broadened arbitrarily by overcoupling them, at the expense of decreasing significantly the photon conversion efficiency due to the reduced intra-cavity optical pump power. An alternative is to selectively over-couple only the optical resonance where the upconverted signal lies, while keeping the pump nearly critically coupled. Mode-selective overcoupling could be achieved by using either polarization-sensitive or resonant coupling structures whose coupling strength is polarization or frequency dependent^25^.
